# CDC’s Guiding Principles to Promote an Equity-Centered Approach to Public Health Communication

**DOI:** 10.5888/pcd20.230061

**Published:** 2023-07-06

**Authors:** Renee M. Calanan, Michelle E. Bonds, Sara R. Bedrosian, Susan K. Laird, Delight Satter, Ana Penman-Aguilar

**Affiliations:** 1 National Center for Emerging and Zoonotic Infectious Diseases, Centers for Disease Control and Prevention, Atlanta, Georgia; 2 Global Health Center, Centers for Disease Control and Prevention, Atlanta, Georgia; 3 Office of Communication, Centers for Disease Control and Prevention, Atlanta, Georgia; 4 Office of Tribal Affairs and Strategic Alliances, Centers for Disease Control and Prevention, Atlanta, Georgia; 5 Member of the Confederated Tribes of Grand Ronde; 6 Office of Health Equity, Centers for Disease Control and Prevention, Atlanta, Georgia

## Abstract

A public health practitioner’s mission is to protect and promote the health of all people in all communities. Components of being successful in that mission include understanding who is at risk of negative outcomes, identifying effective actions to promote and protect health, and communicating information accordingly. Information must be scientifically rigorous, provide appropriate contextualizing information, and refer to and visually represent people through words and images in respectful ways. Public health communication objectives include that the audience accepts, understands, and acts on the information to protect and promote health. This article describes the impetus for, development of, and public health applications and implications of principles to guide communication efforts. CDC’s Health Equity Guiding Principles for Inclusive Communication is a web-based resource published in August 2021 that offers — but does not mandate — guidance and recommendations for public health practice. The resource can help public health practitioners and their partners consider social inequities and diversity, think more inclusively about the people they serve, and adapt to the cultural, linguistic, environmental, and historical situation of each population or audience of focus. Users are encouraged to have conversations about the Guiding Principles as they plan and develop communication products and strategies in collaboration with communities and partners and build a shared vocabulary consistent with how communities and groups of focus see and understand themselves, because words matter. As the public health field renews its focus on shifting the paradigm toward equity, a language and narrative shift is a vital intervention.

**Summary**

**What is already known on this topic?**

Public health services to protect and promote the health of all people involve equity-centered approaches and communication to inform people about factors that influence health and how to improve it.

**What is added by this report?**

This report describes the development of CDC’s Health Equity Guiding Principles for Inclusive Communication and summarizes equity-centered best practices for public health communication.

**What are the implications for public health practice?**

Public health practitioners can apply these principles across their work with collaborative approaches by using respectful language and narrative that might contribute to reducing health inequities.

## Background

Public health practitioners work to ensure that policies, systems, and public health practices enable optimal health and safety for all people in all communities. This work is conducted across federal, state, tribal, local, territorial, and freely associated state public health levels and in collaboration with partners. One of the 10 essential public health services (core public health practices) is to communicate effectively to inform and educate people about health, factors that influence it, and how to improve it (1). Effective communication informs the public, health care providers, public health practitioners, communities, and partners from other sectors to approach the health of all communities in ways that can reduce risks and improve health and safety. An equity-centered approach to inclusive communication — which is respectful communication that uses shared terminology and narrative consistent with how the intended audiences see and understand themselves — can reach more people and therefore be more effective (2). Such narratives are collections of messages and stories that represent values of fairness and justice and describe strengths as well as inequities, their causes, and solutions (3). All people should be able to access and understand health promotion and disease prevention information without stigmatization of themselves or others (4). Public health practitioners have an ethical obligation as well as sound practical reasons to share scientific data results and recommendations that appropriately frame social and health inequities (5). Additionally, practitioners need to make every effort to avoid the continuation of harmful stereotypes. Stigmatizing language can harm people by influencing people’s judgments, including those that affect medical treatment (6). Conversely, inclusive language could contribute to reducing health inequities and increasing opportunities to become as healthy as possible.

The Centers for Disease Control and Prevention (CDC) is committed to advancing health equity. The agency has prioritized integrating equity into all science and intervention strategies and has declared racism a serious threat to the public’s health (7,8). The agency defines health equity as the state in which everyone has a fair and just opportunity to attain their highest level of health (9). Efforts to advance health equity correspond with and are central to core practices needed to accomplish the public health mission successfully. Achieving equity requires

- sustaining focused and ongoing societal efforts to address historical and contemporary injustices among groups that have been marginalized, such as racialized minority groups, people who identify as LGBTQIA+ (lesbian, gay, bisexual, transgender, queer [or questioning], intersex, asexual [or allied]), people living with a disability, and people living in rural areas;
- overcoming economic, social, and other obstacles to health and health care; and
- working to eliminate preventable health disparities.

Long-standing inequities exist among populations that have been historically marginalized, and social, economic, and environmental inequities have substantial effects on health (10). CDC has long understood that social and health inequities challenge the agency’s ability to reach its goals. The COVID-19 pandemic brought increased awareness of these inequities, resulting in a greater push for public health communication about inequities with appropriate, unbiased contextualization and language. The pandemic pushed CDC and many others to counteract social stigma systematically, with communication being an integral component. Improved communication is only one facet of addressing inequities, and it is only one of many steps that CDC is taking to renew its commitment to advancing health equity. CDC invites all public health practitioners to make a renewed commitment to inclusively consider the people it serves and apply an equity-centered public health approach, including communication.

## Development and Dissemination of a Communication Resource

CDC’s Health Equity Guiding Principles for Inclusive Communication (11) was developed in 2 phases. The first phase began in the early stages of CDC’s COVID-19 pandemic response. CDC first established a Chief Health Equity Officer unit (CHEO) for this emergency response structure, in part because of the pandemic’s devastating effects on communities that have historically been stigmatized or excluded. In 2020, CHEO led the development of CDC’s COVID-19 Response Health Equity Strategy (12) and was tasked to review scientific and health promotion products before dissemination. Reviews focused on health equity science, scientific integrity, adherence to CDC policy, and equity-centered communication. These reviews applied both health equity science and health communication science principles to acknowledge the social, cultural, economic, and environmental contexts of health inequities. Given that CHEO was stood up (ie, initiated and established) by CDC’s Office of Health Equity (OHE, formerly Office of Minority Health and Health Equity), the review process was substantially influenced by OHE’s practices for contextualizing data results and addressing stigma and implicit bias in public health science communication. Though guidance had been shared informally with individuals and writing groups across CDC, these practices had not yet been collated and systematized for the agency.

The heightened national consciousness of the persistent, disproportionate risks experienced by certain communities identified an urgent need for a resource that would guide CDC staff participating in the COVID-19 emergency response when developing scientific and other communications (eg, health education, social media). CHEO staff worked with units across the response structure to gather input and resources, including the Community Mitigation Task Force’s draft list of preferred terminology. The initial draft of the resulting COVID-19 Health Equity Style Guide included a review of equity-centered communication science and best practices from peer-reviewed and gray literature and contributions and reviews from numerous CDC subject matter experts. At that time, it was intended as a resource for CDC staff participating in the agency’s COVID-19 response, and as such, it was disseminated internally through response communications, intranet sites, presentations, and meeting discussions. Uptake was strong, and the resource was informally shared with CDC staff who were not participating in the COVID-19 response. Demand clearly existed for this type of resource.

The second phase of development involved refinement and a broader perspective that was not focused on COVID-19. The goal was to create a public-facing resource available for all public health practitioners and partners to apply an equity-centered approach to communication. A CDC work group conducted further review of the content with additional consultation of the literature, subject matter experts, and people with lived experience. After the work group refined and added content, numerous diverse CDC subject matter experts and external partners provided input through rounds of collaborative feedback and revisions before making the guide final. Launch of the Health Equity Guiding Principles for Inclusive Communication website (11) included a presentation for public health communicators at the 2021 National Conference on Health Communication, Marketing and Media (13). CDC and partners broadly disseminated information about the new resource through email, newsletters, websites, social media, and presentations.

Since the launch of the public-facing Guiding Principles website, more than 35 webinars and trainings on the content have been made to almost 5,000 staff members of CDC, National Institutes of Health/National Institute on Aging, the Guide to Community Preventive Services (the Community Guide), academic departments of public health, multiple state and local public health departments, the Impact Assessment Agency of Canada, the American Medical Association (AMA), Association of American Medical Colleges, Merck, and other organizations. Demand for such presentations continues. Subject matter experts and communication staff also provide consultations to groups across CDC who are interested in learning more about applying the Guiding Principles to their work. Additionally, the website provides an email address for questions and feedback about the content. Together with feedback from the presentations, trainings, and consultations, CDC staff review feedback and consider whether revisions should be made to the resource to either clarify, remove, or add content. An annual review of the content also helps to ensure that the content is aligned with the latest science and cultural and social norms, and that it is in accord with related agency resources such as CDC’s Global Public Health Equity Guiding Principles for Communication, which was launched in 2022 (14). AMA incorporated content from the Guiding Principles into its Advancing Health Equity: A Guide to Language, Narrative and Concepts (3), and other organizations have since created resources (15).

## Description of the Resource

The Guiding Principles is a website that covers 2 wide-ranging considerations when developing a communication product: understand and frame the context of the information in terms of social and health inequities (Box 1) and apply best practices for language and images (Box 2). In other words, communicators should use both context and language to create health communication messages that can be heard, understood, and acted on. Again, effective communication is respectful, inclusive, and nonstigmatizing. Communication about inequities must use an approach that appropriately frames data and information in a way that considers the underlying societal factors influencing inequities and methods to prevent exacerbation and eliminate them most effectively.

**Box 1. Applying Key Concepts for Equity-Centered, Inclusive Communication**

**Table:** 

Health equity concept	How to incorporate the concept
Long-standing systemic social and health inequities have put some population groups at increased risk of getting sick, having overall poor health, and having worse outcomes when they do get sick.	• Understand how policies, programs, practices, services, and environments that support health can reduce health inequity (16). • Avoid implying that a person, community, or population is responsible for increased risk of adverse outcomes. • Avoid perpetuating health inequities in communication by considering how racism (8) and other systems of power differentially advantage people.
Diversity exists within and across communities and can be defined by several factors.	• Understand that there is diversity within communities and members of population groups are not all the same in their health and living circumstances. • Limit use of the terms minority and minorities, in general. Refer to groups with an appropriate and relevant level of specificity.
Individuals and communities vary in history and lived experiences, cultural traditions, religious beliefs and practices, social norms, available resources, and many other factors.	• Seek to understand the intended audience to avoid misinformation, errors, confusion, or the loss of credibility. • Adjust recommendations that might not make sense for specific situations, places, communities, or cultures. • Understand that not everyone has access to medical and mental health care or services — including barriers such as lack of insurance, transportation, childcare, and paid work leave — and trust in medical professionals may be limited. • Understand that people may not have full control over their work environment or conditions, and that an employer’s responsibility to provide certain resources or allow certain conditions for workers may vary.
Interconnected structures and systems can create inequality among groups based on social categories (17).	• Be cautious in generalizing about a community. Consider how people’s social identities overlap to better understand, interpret, and communicate about health. • Consider multifaceted approaches to address overlapping connections of individuals and groups with structures and systems that create social and health inequities as well as to leverage strengths and assets.
Achieving health equity requires focused and ongoing societal efforts to address historical and contemporary injustices; overcome economic, social, and other obstacles to the best health and health care; and eliminate preventable health disparities (9).	• Consider that communicating effectively and equitably — to inform and educate about health, factors that influence it, and how to improve it — is an essential public health service (1). • Intentionally consider the potential positive and negative impacts of proposed messages, including how messages could help reduce or contribute to inequities. • Address and refer to people and groups inclusively, respectfully, and accurately. Avoid dehumanizing language.

**Box 2. Strategies for an Equity-Centered Approach to Developing Public Health Communication**

**Table:** 

Strategy	Implementation considerations
Build and support a diverse and skilled public health workforce.	• Build a diverse and inclusive workforce throughout all levels, including leadership positions. • Consider hiring people from the communities served, including disproportionately affected communities, and who look and sound like the communities served. • Ensure capacity to work with community partners to identify priorities and strategies and build community awareness and acceptance before communication products are developed and released. • Promote open discussion of health equity concepts and use of equity-centered communication strategies.
Incorporate meaningful community engagement (18) as a foundational component throughout the process to develop culturally relevant, unbiased communication for health promotion, research, or policy making.	• Remember that successful community engagement is a continuous process that builds trust and relationships through multidirectional communication processes. • Start with mindfulness and listening and continue with joint decision making and shared responsibility for outcomes.
Ensure that public health programs, policies, and practices recognize and reflect the diversity of the community they are trying to reach.	• Ensure that information is culturally responsive (19), represents people in the communities for whom it is intended, and is accessible and available. • Tailor interventions based on the unique circumstances of different populations. Recognize that some members of your audience of focus may not be able to follow public health recommendations because of their cultural norms, beliefs, practices, available resources, or other reasons. • Translate materials into the preferred languages of the intended audience, and make sure a native speaker reviews translated materials. • Work with community members, leaders, and population-specific representatives or experts to develop culturally responsive content. • Emphasize positive actions and highlight community strengths and solutions.
Use clear communication and plain language while recognizing that the audience of focus may not all have the same level of literacy and, specifically, health literacy (20–23).	• Consider both reading level and ability to understand the content in the language presented. • Use active verbs, plain language, and accessible channels and formats so that all members of your audience can access and understand the information. • Avoid jargon and use straightforward, easy to understand language.
Ensure that any images support and do not detract from your message.	• Consider the intended audience, the intended use, and the full set of images planned. • Include members or representatives of your intended population of focus in the decision-making process. • Decide whether an image is culturally appropriate, clear, and inclusive for diverse audiences. • Depict positive and health-promoting behaviors, and don’t unintentionally reinforce stereotypes or perpetuate health inequities. • Include accurate depictions of people within the given context. For example, use accurate depictions of people with a disability and their assistive technology and avoid inappropriate depiction of cultural dress or activity in a daily life setting. • Include alternate text that can be easily understood and images with enough color contrast for people with low visual acuity.

The Guiding Principles is a starting point and an approach, not a mandate, for public health practitioners and partners to intentionally consider in all types of communication. Using an inclusive process with community and partner engagement, practitioners can use this equity-centered approach to tailor and enhance reach and understanding of health information with the ultimate goal of improving health for all people. The 6 sections of the website are described below.

- **Using a health equity lens:** This section emphasizes that public health programs, policies, and practices are more likely to succeed when they recognize and reflect the diversity of audiences they are trying to reach. It describes actions to intentionally assess potential positive and negative impacts of proposed messages and to consult and collaborate with groups from intended audiences to reach those audiences most effectively. It recognizes intersectionality (17) and the need to understand the overlapping individual and systems-level contexts that create inequality based on social categories (eg, race, class, gender), as well as communities’ unique assets and influences.
- **Key principles: **This section lists several key principles, including avoiding terms that are inadequately specific or imply a condition is the fault of a specific group, using person-first language to intentionally recognize humanity, limiting use of the term minority or minorities, avoiding language with violent connotations, and avoiding blaming and stigmatization in how people’s actions, inactions, or conditions are described.
- **Preferred terms:** This section provides suggestions for terms that could be used to increase inclusiveness and decrease blaming and stigmatization. It is meant to be used as a guide and inspiration to learn more and engage people from the population or community of focus to understand their preferences. The section is not comprehensive — the listed terms are not intended to be the only terms to avoid or use to improve messages.
- **Developing inclusive communications: **This section provides suggestions for developing public health communications related to specific topics, including images, cultural responsiveness, appropriateness of public health guidance for an intended audience, disability (24,25), mental and behavioral health (26), and older adults (27).
- **Inclusive images:** This section provides detailed suggestions for selecting culturally appropriate, inclusive photographs or images for health communication materials, including considering the intended audience, the intended use of the image, how it supports the communication, and in what format the images will be disseminated.
- **Resources and references: **This section provides selected resources and best practices for inclusive language and framing health inequities, many of which were used as resources in the development of the Guiding Principles.

For more information, see CDC’s Health Equity Guiding Principles for Inclusive Communication website, https://www.cdc.gov/healthcommunication/Health_Equity.html.

## Applications in Public Health Practice

CDC encourages all public health practitioners to identify opportunities to apply these Guiding Principles across all their work, such as when engaging with communities, partners, and colleagues and when developing scientific publications and recommendations (Box 3). The resource is designed to be used throughout planning, development, writing, and dissemination of communication products. The Guiding Principles can be used in epidemiology and surveillance, program planning, evaluation, policy, and other essential public health functions.

**Box 3. Examples of CDC’s Experiences in Applying and Discussing Health Equity Guiding Principles for Inclusive Communication**

**Scientific Practice Example**

Recently, a national group of American Indian and Alaska Native experts from multiple fields were charged with writing a complex scientific primer, *American Indian and Alaska Native Knowledge and Public Health for the Primary Prevention of Missing or Murdered Indigenous Persons* (28), for a nonpublic health audience, with a 3-week turnaround. The authors had multiple goals for the paper, which included bringing prominence to tribal elders’ traditional knowledge to complement public health science, epidemiology, psychology, and the law. The writing group was challenged with shared intentionality, defining communication goals upfront, and in the end, respecting diverse views, while presenting a unified voice for the reader. There were no challenges with purpose, goals, or cooperation.

All authors were subject matter experts and had collectively authored thousands of books, papers, health education materials, policies, and laws. The authors needed a process to ensure their language was inclusive and contemporary for a primary audience of legal scholars, judges, and law enforcement. For speed, they broke into teams and wrote sections based on their scientific and practice experience, then met to review and negotiate challenges. During this review process, they used the Guiding Principles as a practical tool to eliminate jargon, evaluate habitual language, and improve the writing.

**Public Health Communication Example**

Since the launch of the Guiding Principles, more than 35 presentations to nearly 5,000 listeners have been provided to a diverse group of people both internal and external to CDC. The approach used introduces the core concepts of the Guiding Principles, using an invitational versus a mandated approach to join in the work of being more inclusive. The idea of “meeting people where they are” recognizes that although the audiences are primarily public health professionals, each listener brings a different world view. The presenters acknowledge this fundamental concept and address issues of racism, ageism, generational influences, cultural influences, and intersectionality to highlight the importance of understanding that change is a process and the ability to view the world inclusively through an equity-focused lens requires continuous learning.

The Guiding Principles have received both positive feedback and pushback, and the authors recognize that there is much work to be done. Using the invitational approach has encouraged people to speak freely about their responses to the concepts. Questions often include requests for justification for the suggested terminology as well as requests and suggestions for the addition of terms that have not yet been included in the work.

As a result of these presentations, people have revealed their personal challenges with this work. One person who self-identified as a middle-aged, White man noted that he felt like he was overly cautious because of the attention on the subject. He said that he was self-conscious about speaking out in meetings now for fear he might say the wrong thing. Others have questions about why terms such as “target population” and “stakeholder” are now considered offensive when they have been used for many years. The authors recognize and support that this process will take time, patience, and open minds to be successful. Continued discussions of the Guiding Principles are critical to our collective learning.

The Guiding Principles are founded on respect for diversity and inclusion. Products are more effective if authors incorporate diverse input by using inclusive engagement of the intended audience. The first step in developing any communication product is to identify and consider the intended audience. Engaging people from the population or community of focus for input and terminology preferences is a best practice. The bottom line is: it is important to know your audience. Authors (public health practitioners) do not get to decide what is stigmatizing to someone else or a community. And when key issues important to that audience are not addressed, this omission could negatively influence the goals of the public health program. What is left unsaid (eg, not providing context about underlying causes of health inequities) can put the burden on the audience to make those connections and could lead to inaccurate interpretations and takeaways from the communication.

The Guiding Principles provides a starting point to improve public health writing and communication. Language and culture are both dynamic and shift across the years and generations, regionally and within population groups. The Guiding Principles are not meant to mandate language in health communication, but rather are a tool for further thought, information collection, community and partner engagement, and data analysis and interpretation. In selecting terms to be used to refer to specific population groups or communities, the Guiding Principles are not prescriptive.

Public health practitioners can refer to the Guiding Principles when answering the following questions to take an equity-centered approach to their work:

1. How do social and health inequities influence the topic?
2. How should planning and implementation of the public health activity be responsive to the inequities?
3. Will (or does) the activity perpetuate existing inequities?
4. How can the Guiding Principles be applied to improve communication and meet the public health needs of the communities served?
5. Being mindful that language, culture, and norms are dynamic, how can we commit to enhancing and maintaining learning, awareness, and humility to improve communication?

## Strengths and Limitations of the Resource

The Guiding Principles is designed to be a living resource that will be updated as culture, norms, and language evolve and the associated science and evidence base grow. The resource is updated periodically and at least annually (eg, content was recently added about images). Users of the resource are encouraged to bookmark the website and refer to it often, as updates are made periodically.

The routine updates to this resource and active dissemination through training and discussions are meant to promote continued learning and more effective communication. It intends to help people understand that words and images matter — they can either support inclusiveness through an equity-centered approach or reinforce harmful stereotypes and marginalization. The resource includes current best practices toward an equity-centered approach, including that being effective in that approach cannot be realized in isolation, though further evaluation of these practices is needed. Meaningful community engagement is key to growth and learning (29).

A potential limitation of the Guiding Principles is that they can be misinterpreted as a directive style guide, as opposed to an intentional approach with suggested terminology to consider. Principles and preferred terms should be considered in each specific context (eg, type of product, audience, population-specific focus). In addition, some terms might not always be appropriate or inappropriate, depending on context and audience, and any potential unintended outcomes (eg, alienation of another group) should be assessed. It is also not comprehensive — every possible consideration, topic, or population of focus is not included. The reader should identify how to apply the principles to any additional areas by using equity-centered, inclusive approaches outlined in the resource.

Some common health equity science considerations (eg, choice of an analytic comparison group) are beyond the scope of what could be addressed in the communication product development process, and those are being incorporated in ongoing CDC efforts to elevate and systematize equity-focused scientific best practices. In addition, the Guiding Principles cannot fix foundational problems in public health science, program, or activity approaches. For example, a poorly designed study or a poorly implemented program or activity cannot be fixed with words.

The lack of an evaluation of the resource means that we cannot yet determine the effectiveness of applying the Guiding Principles. The authors are aware that numbers of people reached with presentations and the volume of hits to the website do not represent agreement with the concepts or use of the principles, again reflecting the importance of continuing to review, reflect, and update as language and culture evolves. Equally important is continuing to engage in discussions about the principles with colleagues and partners, evaluating the process and outcomes of efforts to disseminate and apply the principles, and contributing to the development and refinement of best practices.

## Population Health and Health Equity

The Guiding Principles was developed and disseminated during a divisive time of social conflict, misinformation, and mistrust of public health, but this is not a new problem. Public health practitioners need to consider this continually challenging environment when communicating with a diverse public. Recognizing that perspectives and opinions differ, including among public health practitioners, will help in planning and implementing public health activities effectively. For example, understanding the values, beliefs, and experiences that lead intended audiences to trust or mistrust sources of information will help practitioners to craft communication products with messages that resonate and to disseminate those messages through appropriate channels. Humility and openness to new perspectives and changing language and norms may improve effectiveness, ensure responsiveness to communities, and help inform decisions that promote health for all.

When social and health inequities are addressed, this benefits all people and overall population health (30). Equity-centered public health approaches must be systematic and multifaceted. Communication is simply one set of tools in the toolbox, as it is only 1 of the 10 essential public health services. Communication efforts that use the right tools get better results. Public health practitioners must work across disciplines and with diverse colleagues and partners to achieve the vital goal of health equity. For example, ensuring collaboration among a diverse and representative team of communicators, scientists, statisticians, policy experts, and partners throughout the life cycle of a public health activity may ensure stronger and more effective communication and public health outcomes. Public health practitioners must recognize that they are all communicators and should continually reflect on the effects of their words (and actions). Building trust and being respectful is both an individual and collective effort that is essential in protecting and promoting health and well-being for all.

## References

[1] Centers for Disease Control and Prevention. 10 Essential public health services. 2023. Accessed February 28, 2023. https://www.cdc.gov/publichealthgateway/publichealthservices/essentialhealthservices.html

[2] Boston University Medical Group, Office of Equity, Vitality, and Inclusion, and BUMC Faculty Development and Diversity. Inclusive language practices. 2022. Accessed February 28, 2023. https://www.bumc.bu.edu/bumg/files/2021/10/Inclusive-Language-Practices_101821.pdf

[3] American Medical Association. Association of American Medical Colleges. Advancing health equity: a guide to language, narrative and concepts. 2021. Accessed February 28, 2023. https://www.ama-assn.org/system/files/ama-aamc-equity-guide.pdf

[4] Smith RA , Applegate A . Mental health stigma and communication and their intersections with education. Commun Educ 2018;67(3):382–93. 10.1080/03634523.2018.1465988 31354181 PMC6660176

[5] Chukwumerije N . Equitable health care requires inclusive language. Harvard Business Review. 2022. Accessed February 28, 2023. https://hbr.org/2022/07/equitable-health-care-requires-inclusive-language

[6] Kelly JF , Saitz R , Wakeman S . Language, substance use disorders, and policy: the need to reach consensus on an “addiction-ary”. Alcohol Treat Q 2016;34(1):116–23. 10.1080/07347324.2016.1113103

[7] Centers for Disease Control and Prevention. CDC CORE Health Equity Science and Intervention Strategy. 2022. Accessed February 28, 2023. https://www.cdc.gov/healthequity/core/index.html

[8] Centers for Disease Control and Prevention. Racism and health. 2021. Accessed February 28, 2023. https://www.cdc.gov/minorityhealth/racism-disparities/

[9] Centers for Disease Control and Prevention. Health equity. 2023. Accessed February 28, 2023. https://www.cdc.gov/healthequity/index.html

[10] Jack L Jr . Advancing health equity, eliminating health disparities, and improving population health. Prev Chronic Dis 2021;18:E79. 10.5888/pcd18.210264 34387187 PMC8388204

[11] Centers for Disease Control and Prevention. Health equity guiding principles for inclusive communication. 2022. Accessed February 28, 2023. https://www.cdc.gov/healthcommunication/Health_Equity.html

[12] Centers for Disease Control and Prevention. CDC COVID-19 response health equity strategy: accelerating progress towards reducing COVID-19 disparities and achieving health equity. 2022. Accessed February 28, 2023. https://www.cdc.gov/coronavirus/2019-ncov/community/health-equity/cdc-strategy.html

[13] National Conference on Health Communication, Marketing & Media. Health equity guiding principles for inclusive communication — making it stick. 2021. Accessed February 28, 2023. https://www.nchcmm.org/index.php/healthequity

[14] Centers for Disease Control and Prevention. Global public health equity guiding principles for communication. 2022. Accessed February 28, 2023. https://www.cdc.gov/globalhealth/equity/guide/index.html

[15] CommunicateHealth, Inc. A framework for equity-centered health communication. 2023. Accessed March 9, 2023. https://communicatehealth.com/wp-content/uploads/CH-ECHC-Framework.pdf

[16] Centers for Disease Control and Prevention. Social determinants of health at CDC. 2022. Accessed March 24, 2023. https://www.cdc.gov/about/sdoh/index.html

[17] Crenshaw K . Mapping the margins: intersectionality, identity politics, and violence against women of color. Stanford Law Rev 1991;43(6):1241–99. 10.2307/1229039

[18] Centers for Disease Control and Prevention/Agency for Toxic Substances and Disease Registry. CDC/ATSDR principles of community engagement. Second edition. 2015. Accessed February 28, 2023. https://www.atsdr.cdc.gov/communityengagement/

[19] US Department of Health and Human Services, Office of Minority Health. Think cultural health. 2021. Accessed February 28, 2023. https://minorityhealth.hhs.gov/omh/browse.aspx?lvl=2&lvlid=53

[20] Centers for Disease Control and Prevention. Health literacy. 2022. Accessed February 28, 2023. https://www.cdc.gov/healthliteracy/index.html

[21] US Department of Health and Human Services, Office of Disease Prevention and Health Promotion. Health literacy and health equity: connecting the dots. 2021. Accessed February 28, 2023. https://health.gov/news/202110/health-literacy-and-health-equity-connecting-dots#:~:text=Health%20literacy%20principles%20make%20information,in%20their%20public%20health%20interventions

[22] Centers for Disease Control and Prevention. The CDC Clear Communication Index. 2021. Accessed February 28, 2023. https://www.cdc.gov/ccindex/index.html

[23] Public Health Communications Collaborative. Communications tool: plain language for public health. 2023. Accessed February 28, 2023. https://publichealthcollaborative.org/resources/plain-language-for-public-health/

[24] Centers for Disease Control and Prevention, National Center for Birth Defects and Developmental Disabilities. Communicating with and about people with disabilities. 2022. Accessed February 28, 2023. https://www.cdc.gov/ncbddd/disabilityandhealth/materials/factsheets/fs-communicating-with-people.html

[25] National Association of the Deaf. Community and culture – frequently asked questions. 2023. Accessed February 28, 2023. https://www.nad.org/resources/american-sign-language/community-and-culture-frequently-asked-questions

[26] American Psychological Association. Bias-free language. 2023. Accessed February 28, 2023. https://apastyle.apa.org/style-grammar-guidelines/bias-free-language/

[27] Centers for Disease Control and Prevention. Underlying medical conditions associated with higher risk for severe COVID-19: information for healthcare professionals. 2023. Accessed February 28, 2023. https://www.cdc.gov/coronavirus/2019-ncov/hcp/clinical-care/underlyingconditions.html 34009770

[28] Satter DE , Mercer Kollar LM ; Public Health Writing Group on Missing or Murdered Indigenous Persons Various Public Health Experts; O’Gara ‘Djik Sook’ D . American Indian and Alaska Native knowledge and public health for the primary prevention of missing or murdered Indigenous persons. Dep Justice J Fed Law Pract 2021;69(2):149–88. 34734212 PMC8563020

[29] Organizing Committee for Assessing Meaningful Community Engagement in Health and Health Care Programs and Policies. Assessing meaningful community engagement: a conceptual model to advance health equity through transformed systems for health. National Academy of Medicine; 2022. 10.31478/202202c PMC9303007 35891775

[30] National Academies of Sciences, Engineering, and Medicine; Health and Medicine Division; Board on Population Health and Public Health Practice; Committee on Community-Based Solutions to Promote Health Equity in the United States; Baciu A , Negussie Y , Geller A , Weinstein JN , editors. The root causes of health inequity. In: Communities in action: pathways to health equity. National Academies Press; 2017. https://www.ncbi.nlm.nih.gov/books/NBK425845/ 28418632

